# Perioperative Hydrogen Inhalation and Postoperative Pain After Endoscopic Discectomy: A Pilot Non-Randomized Clinical Study with Exploratory Murine Data

**DOI:** 10.3390/medicina62091785

**Published:** 2026-09-16

**Authors:** Chao-Hsien Sung, Wen-Chin Ko, Chia-Chi Kung, Chi-Feng Hung

**Affiliations:** 1 Division of Anesthesiology, Fu Jen Catholic University Hospital, Fu Jen Catholic University, New Taipei City 24352, Taiwan; a02664@mail.fjuh.fju.edu.tw; 2 Ph.D. Program in Pharmaceutical Biotechnology, Fu Jen Catholic University, New Taipei City 242062, Taiwan; 3 School of Medicine, Fu Jen Catholic University, New Taipei City 242062, Taiwan; 086938@mail.fju.edu.tw; 4 Division of Cardiac Electrophysiology, Department of Cardiovascular Center, Cathay General Hospital, Taipei 10630, Taiwan; 5 School of Pharmacy, Kaohsiung Medical University, Kaohsiung 80708, Taiwan

**Keywords:** hydrogen inhalation, postoperative pain, chronic sciatica, endoscopic discectomy, chronic constriction injury, pilot study

## Abstract

*Background and Objectives*: Postoperative pain after endoscopic discectomy may reflect acute surgical nociception superimposed on pre-existing radicular symptoms. Molecular hydrogen (H_2_) has antioxidant and anti-inflammatory properties, but clinical evidence for perioperative analgesia is limited. We evaluated the association between perioperative H_2_ inhalation and postoperative pain and analgesic requirements. *Materials and Methods*: This open-label, non-randomized pilot study enrolled 37 patients in two consecutive periods (H_2_, *n* = 25; control, *n* = 12). Because original outcome records for 12 H_2_-period participants could no longer be retrieved at the time of manuscript preparation, the complete-case clinical analysis included 25 source-verifiable participants (H_2_, *n* = 13; control, *n* = 12). H_2_ was delivered via nasal cannula as a 66.7% H_2_/33.3% O_2_ source gas at 2 L/min (estimated inspired H_2_ approximately 4%). Pain was evaluated with a numerical rating scale (NRS) preoperatively, at post-anesthesia care unit (PACU) arrival, at 1, 6, 12, and 24 h, and at 1 month. Longitudinal scores were analyzed using generalized estimating equations with Holm-adjusted comparisons. A parallel exploratory murine chronic constriction injury (CCI) experiment (*n* = 6/group) assessed mechanical allodynia over 14 days of daily H_2_ inhalation. *Results*: The group-by-time interaction was significant (Wald χ^2^(6) = 56.68, *p* < 0.001). After Holm adjustment, pain scores were lower with H_2_ at PACU arrival (difference, −3.21; adjusted *p* = 0.002), 1 h (−2.42; adjusted *p* = 0.004), and 1 month (−3.45; adjusted *p* < 0.001). Intraoperative fentanyl use and rescue analgesic use were also lower in the H_2_ group; no adverse events were documented in the analyzed cohort. In the murine CCI experiment, H_2_-treated mice showed higher ipsilateral paw withdrawal thresholds than air-exposed controls. *Conclusions*: Perioperative H_2_ inhalation was associated with lower pain scores at selected time points and reduced analgesic requirements. These preliminary findings require confirmation in randomized trials. Trial registration: ClinicalTrials.gov NCT05476575.

## 1. Introduction

Chronic sciatica from lumbar nerve-root compression can cause persistent radicular pain and functional impairment. Endoscopic discectomy can relieve radicular symptoms, but the perioperative period adds acute nociceptive pain from surgical tissue injury [1,2,3,4,5]. Postoperative pain in these patients therefore likely reflects a mixture of acute surgical nociception and pre-existing radicular symptoms rather than a purely neuropathic phenotype.

Inflammation and oxidative stress contribute to both postoperative and neuropathic pain. Tissue and nerve injury increase reactive oxygen species (ROS), pro-inflammatory signaling, and nociceptor excitability, with downstream involvement of nuclear factor kappa-light-chain-enhancer of activated B cells (NF-κB) and related pathways [6,7,8,9,10,11,12,13]. These mechanisms give a rationale for testing interventions that modulate oxidative and inflammatory signaling, while recognizing that clinical postoperative pain is multifactorial.

Molecular hydrogen (H_2_) has been studied as a biologically active gas with antioxidant and anti-inflammatory effects. Experimental work indicates that H_2_ can reduce highly reactive oxidative species and modulate inflammatory signaling, including NF-κB- and NLRP3-related pathways [14,15,16].

Preclinical studies have reported antinociceptive effects of hydrogen-containing interventions in several neuropathic and postoperative pain models [17,18,19,20,21], but clinical evidence for perioperative hydrogen inhalation remains limited. The primary aim of this pilot study was therefore to assess the association between perioperative hydrogen inhalation and postoperative numerical rating scale (NRS) pain scores, intraoperative fentanyl consumption, and rescue analgesic use in patients undergoing endoscopic discectomy. A parallel murine CCI experiment was included as exploratory supporting evidence in a standardized neuropathic pain model.

## 2. Materials and Methods

### 2.1. Study Design Overview

This investigation comprised a pilot, open-label, non-randomized human clinical study in patients undergoing endoscopic discectomy for chronic sciatica and an exploratory murine chronic constriction injury (CCI) experiment. An exploratory analysis of paired preoperative and postoperative peripheral blood mononuclear cell (PBMC) inflammatory responsiveness was available only in hydrogen-treated participants and is therefore presented in the Supplementary Materials as supporting evidence rather than as a mechanistic endpoint of the clinical study. The clinical report follows recommendations for non-randomized pilot and feasibility studies [22], and the animal component is reported with reference to the ARRIVE 2.0 guidelines [23].

### 2.2. Clinical Trial: Participants and Ethical Approval

**Figure 1:**
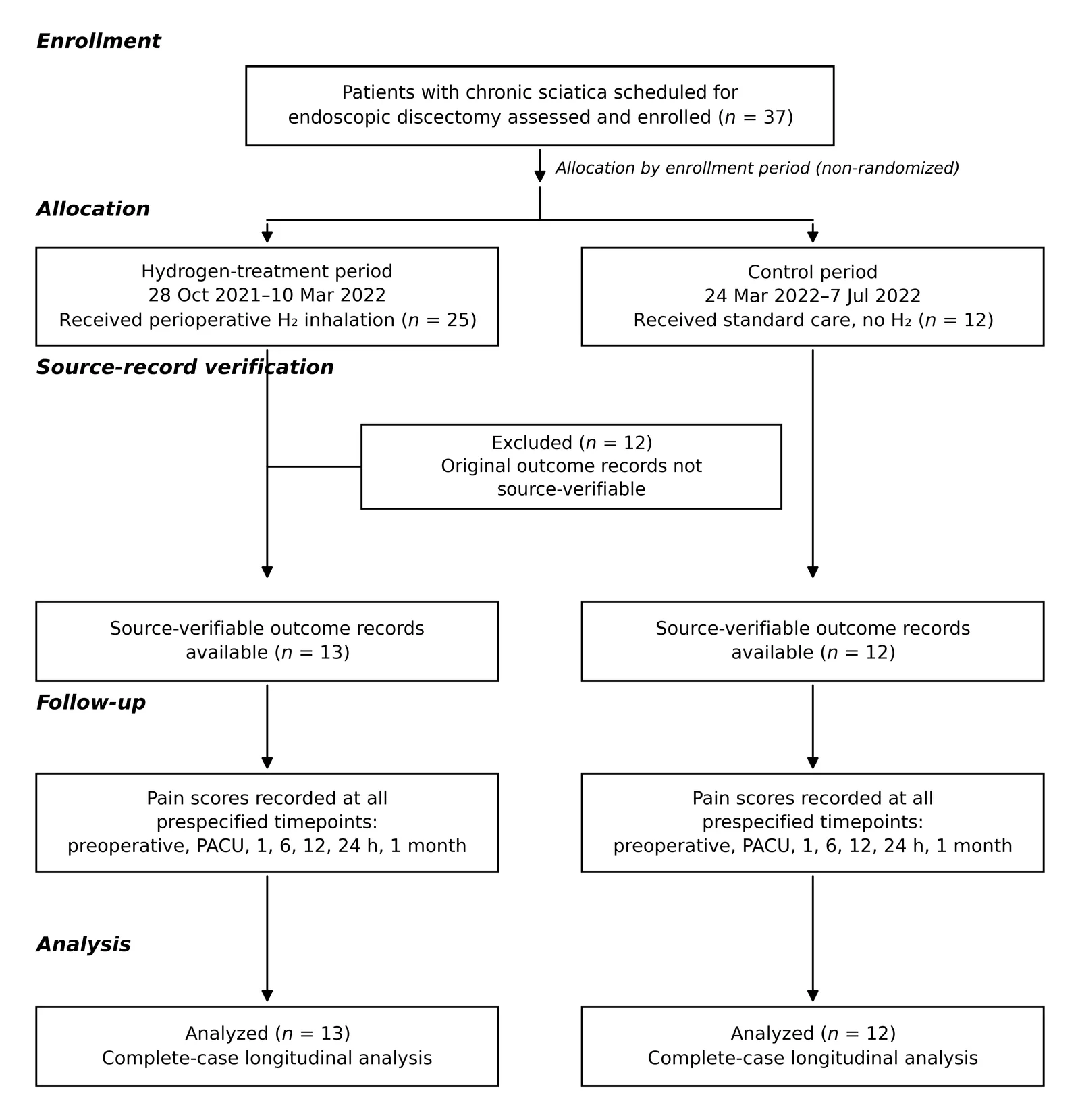
Participant flow. Treatment assignment was determined by two consecutive enrollment periods rather than randomization. Outcome records for 12 participants from the hydrogen-treatment period could not be verified against source documents at the time of manuscript preparation and were excluded from the complete-case analysis.

The study was conducted in accordance with the Declaration of Helsinki and was approved by the Institutional Review Board of Fu Jen Catholic University Hospital (protocol code FJUH110133; 17 September 2021) and prospectively registered at ClinicalTrials.gov (NCT05476575). Written informed consent was obtained before enrollment. Patients with chronic sciatica refractory to conservative treatment who were scheduled for endoscopic discectomy were enrolled in two consecutive study periods: the hydrogen-treatment cohort first (28 October 2021 to 10 March 2022; *n* = 25), followed by the non-hydrogen control cohort (24 March to 7 July 2022; *n* = 12). No randomization or alternating allocation was performed; treatment assignment was determined by the enrollment period. Complete, source-verifiable clinical outcome records were available for 25 patients. Manuscript preparation began more than two years after the study had been completed, and by that time portions of the original study records for 12 patients from the hydrogen-treatment period could no longer be retrieved from the study archive; because reconstructed values could not be verified against source documents, they were not used. Participant flow is summarized in Figure 1. The complete-case analysis therefore included 13 hydrogen-treated patients and 12 controls. Exclusion criteria were (1) American Society of Anesthesiologists (ASA) physical status > IV; (2) severe cardiac, pulmonary, hepatic, or renal dysfunction; (3) autoimmune disease; (4) age < 20 years; (5) age > 75 years; (6) refusal of hydrogen inhalation; (7) pregnancy; and (8) anemia (hemoglobin < 10 g/dL).

### 2.3. Hydrogen Inhalation Protocol (Clinical)

Hydrogen was generated by water electrolysis using a commercial hydrogen generator (model BYT-JP-H03, Shanghai Asclepius Meditec Co., Ltd., Shanghai, China), producing a gas mixture containing 66.7% H_2_ and 33.3% O_2_. The source gas was delivered via nasal cannula at a total flow of 2 L/min throughout the surgical procedure and discontinued at surgical completion. The H_2_ delivery rate was approximately 1.33 L/min (2 L/min × 0.667). The approximate 4% H_2_ concentration specified in the trial registration represented an estimated nominal inspired concentration after dilution with entrained ambient air, rather than the generator outlet concentration. Assuming an inspiratory flow of approximately 30 L/min during spontaneous breathing, the nominal inspired H_2_ fraction was approximately 4.4% (1.33/30) and was rounded to approximately 4% in the protocol. Because low-flow nasal cannula delivery is an open system, actual inspired H_2_ concentration varies with tidal volume, respiratory rate, inspiratory time, mouth breathing, and ambient-air entrainment; inspired H_2_ concentration was not directly measured. The control group received standard oxygen/air via nasal cannula. Patients were maintained under mild-to-moderate sedation (Observer Assessment of Alertness/Sedation score 3–4) using propofol, midazolam, and fentanyl with standardized monitoring for sedation and anesthesia.

### 2.4. Study Outcomes

The primary clinical outcome was postoperative pain intensity recorded on the NRS (0 = no pain, 10 = worst pain) at post-anesthesia care unit (PACU) arrival and at 1, 6, 12, and 24 h and 1 month postoperatively, as prespecified in the IRB-approved protocol. Secondary clinical outcomes included intraoperative fentanyl consumption and rescue analgesic use in the ward. All analyzed patients followed the same postoperative analgesic protocol comprising tramadol, acetaminophen, and non-steroidal anti-inflammatory drugs, with intravenous opioids reserved for breakthrough pain. Exploratory paired PBMC samples (preoperative and postoperative) obtained only from hydrogen-treated participants are described in the Supplementary Materials.

### 2.5. Animal Ethics and Housing

All animal procedures were approved by the Institutional Animal Care and Use Committee (IACUC) of Fu Jen Catholic University (protocol No. A11079) and were conducted in accordance with the 3R principles and ARRIVE 2.0 reporting recommendations [23]. Male C57BL/6 mice aged 8–12 weeks were obtained from BioLASCO Taiwan (Taipei, Taiwan) and housed in groups of 3–5 under a 12-h light/dark cycle with illumination commencing at 07:00 and ad libitum access to food and water. Six mice were analyzed per group. The allocation of rats was not randomized. Only male mice were used in the original experiment, and sex-specific effects were not evaluated.

### 2.6. Chronic Constriction Injury Surgery

Neuropathic pain was induced using the chronic constriction injury (CCI) model described previously [24,25]. Mice were anesthetized with Avertin (20 mg/mL working solution, 0.2 mL per 10 g body weight, intraperitoneally). The right sciatic nerve was exposed at the mid-thigh level, and three loose ligatures of 3–0 silk suture (Unik, New Taipei City, Taiwan) were placed approximately 1 mm apart around the nerve.

### 2.7. Hydrogen Inhalation Protocol (Animal)

Commencing on the day of CCI surgery, mice received daily inhalation of a 66.7% H_2_/33.3% O_2_ gas mixture for 1.5 h within a sealed chamber equipped with an ambient-air aperture for 14 consecutive days. Control mice were placed in identical chambers and exposed to ambient air for the same duration. The control exposure was therefore not oxygen-matched: hydrogen-treated mice inhaled an oxygen fraction of approximately 33.3%, whereas control mice breathed ambient air at approximately 21% oxygen.

### 2.8. Behavioral Assessment

Mechanical allodynia was assessed on days 0 (pre-surgery), 3, 7, and 14 after chronic constriction injury (CCI) by an investigator blinded to group allocation, with testing conducted between 09:30 and 17:00. Mice underwent 2 h of habituation per day for 2 consecutive days before baseline assessment. Von Frey filaments (Touch-Test; North Coast Medical, Morgan Hill, CA, USA) were applied five times at 15 s intervals to the plantar surface of each hind paw. Paw withdrawal threshold (PWT) was defined as the minimum force eliciting withdrawal in more than three of five applications. Both ipsilateral and contralateral paws were assessed.

### 2.9. Statistical Analysis

Continuous clinical variables are summarized as mean ± standard deviation and were compared using two-tailed independent-samples Student’s *t*-tests. Given the small sample, distributional assumptions could not be verified robustly, and these comparisons are descriptive. Sparse 2 × 2 categorical outcomes were analyzed using Fisher’s exact test; the multilevel spinal-level comparison was evaluated using an exact contingency-table procedure. Serial NRS pain scores were analyzed using generalized estimating equations (GEE) [26] with a Gaussian distribution and identity link. Participant ID was specified as the subject variable, assessment time was treated as a categorical within-subject factor, and the model included treatment group, time, and the group-by-time interaction. A working-independence correlation structure was specified together with a robust (sandwich) covariance estimator, and Type III Wald chi-square tests were used for model effects. The group-by-time interaction was used as the principal longitudinal test in the analysis to determine whether the pattern of pain-score change differed between groups. Hydrogen-minus-control contrasts were then estimated from the fitted GEE model at each of the prespecified assessment time points to localize the overall interaction rather than to serve as independent tests of efficacy. Because six postoperative contrasts were examined, their *p* values were adjusted using the Holm step-down procedure [27] to control the family-wise type I error rate. Effect estimates are reported as mean differences with conventional 95% Wald confidence intervals (CIs); these CIs were not multiplicity-adjusted and are presented to describe effect magnitude and precision. The preoperative comparison was not included in the six-comparison postoperative Holm family. At the design stage, a sample-size estimate assuming a 3-point pain-score difference with an anticipated standard deviation of 2.5 indicated that at least 12 patients per group would provide 80% power at alpha = 0.05 for a two-group comparison at a single time point. No power calculation was performed for the group-by-time interaction; the analysis is therefore exploratory and not intended to provide definitive inference. Animal PWT data were analyzed by GEE [26] with a Gaussian distribution and identity link, specifying animal as the subject variable, an exchangeable working correlation structure, and a robust (sandwich) covariance estimator, with ipsilateral and contralateral paws modeled separately. Hydrogen-minus-air contrasts were estimated at each postoperative day and adjusted using the Holm step-down procedure; the day-0 baseline comparison was not included in that family. Statistical significance was defined as *p* < 0.05. The GEE model and other primary clinical analyses were performed using IBM SPSS Statistics for Windows, version 26.0 (IBM Corp., Armonk, NY, USA).

## 3. Results

### 3.1. Cohort Characteristics and Primary Clinical Outcomes

Between 28 October 2021 and 7 July 2022, 37 patients were enrolled: 25 during the hydrogen-treatment period and 12 during the subsequent control period. The final 1-month follow-up included in this analysis was completed on 7 August 2022. Twelve patients from the earlier hydrogen-treatment cohort were excluded from the final clinical analysis because their complete original clinical outcome records could not be reliably retrieved and source-verified during later data consolidation. The complete-case analysis therefore included 25 patients: 13 in the hydrogen group and 12 in the control group. Baseline demographic and clinical characteristics among the analyzed patients were similar between groups (Table 1). No adverse events were documented in the analyzed cohort. Intraoperative fentanyl consumption was lower in the hydrogen group than in controls (265.38 ± 99.24 μg vs. 347.92 ± 93.82 μg; mean difference, −82.54 μg; 95% CI, −162.61 to −2.47; *p* = 0.044). Rescue analgesic use was also less frequent in the hydrogen group (5/13 [38.5%] vs. 10/12 [83.3%]; Fisher exact *p* = 0.041).

### 3.2. Postoperative Pain Scores

**Figure 2:**
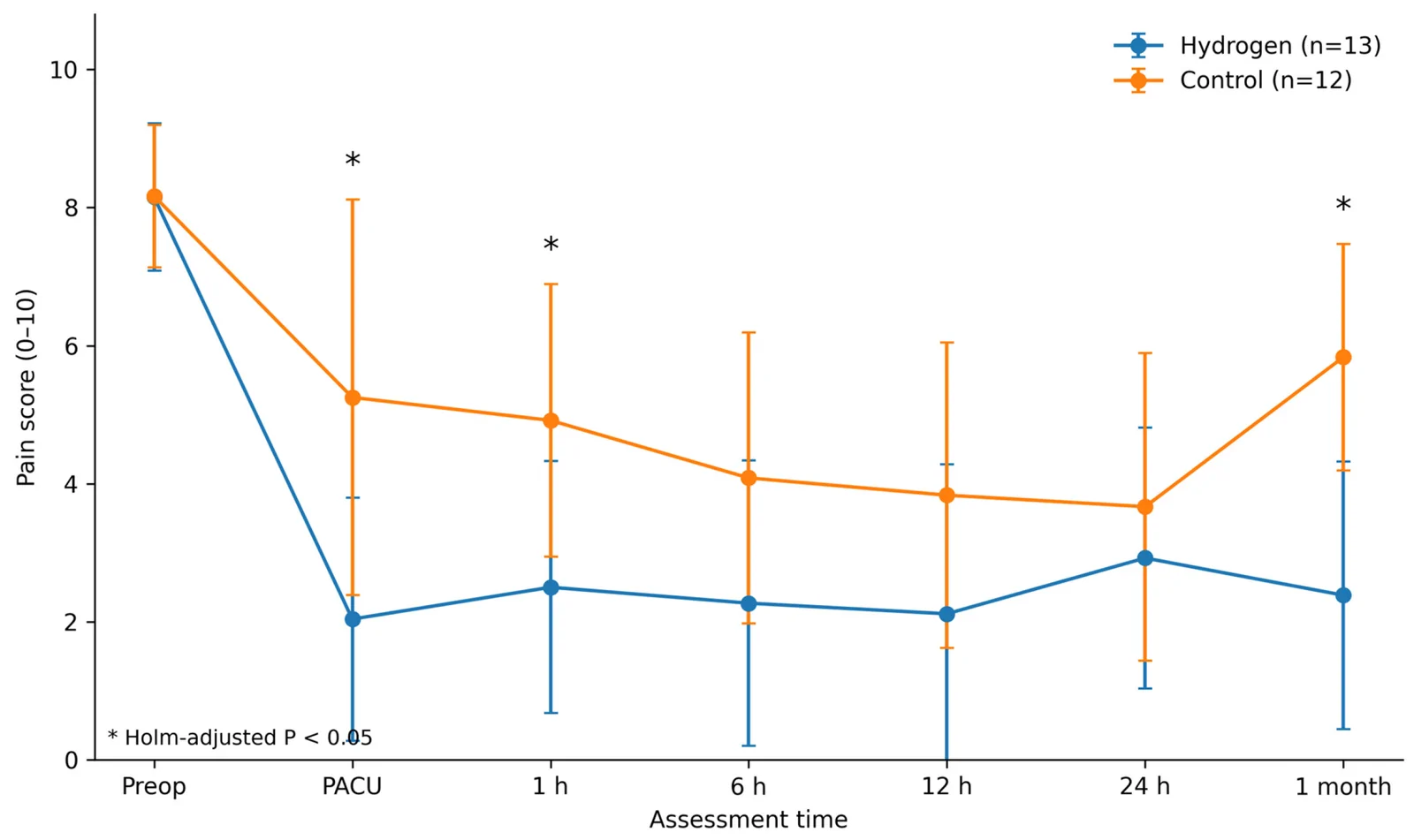
Postoperative NRS pain scores over time in the hydrogen and control groups. Data are presented as mean ± standard deviation. Asterisks indicate postoperative between-group contrasts that remained significant after Holm adjustment (PACU arrival, 1 h, and 1 month). Complete effect estimates, 95% CIs, and adjusted *p* values are shown in Table 2. PACU: post-anesthesia care unit. * Statistically significant (*p* < 0.05).

The GEE analysis included all 175 repeated observations from the 25 analyzed participants (13 hydrogen and 12 control). Preoperative pain scores were similar between groups (8.15 ± 1.07 vs. 8.17 ± 1.03; GEE-estimated hydrogen-minus-control mean difference, −0.01; 95% CI, −0.80 to 0.78; *p* = 0.975) (Figure 2). The group-by-time interaction was significant (Wald χ^2^(6) = 56.68, *p* < 0.001), indicating that the longitudinal pattern of pain-score change differed between groups. After Holm adjustment for the six postoperative contrasts, statistically significant between-group differences remained at PACU arrival (mean difference, −3.21; adjusted *p* = 0.002), 1 h (−2.42; adjusted *p* = 0.004), and 1 month (−3.45; adjusted *p* < 0.001), but not at 6, 12, or 24 h. Complete descriptive means, GEE-derived effect estimates, 95% Wald CIs, and Holm-adjusted *p* values are provided in Table 2. The reported 95% CIs are conventional, non-multiplicity-adjusted intervals; therefore, exclusion of zero by the 6 and 12 h CIs is not inconsistent with their Holm-adjusted *p* values exceeding 0.05.

### 3.3. Exploratory Murine CCI Model

**Figure 3:**
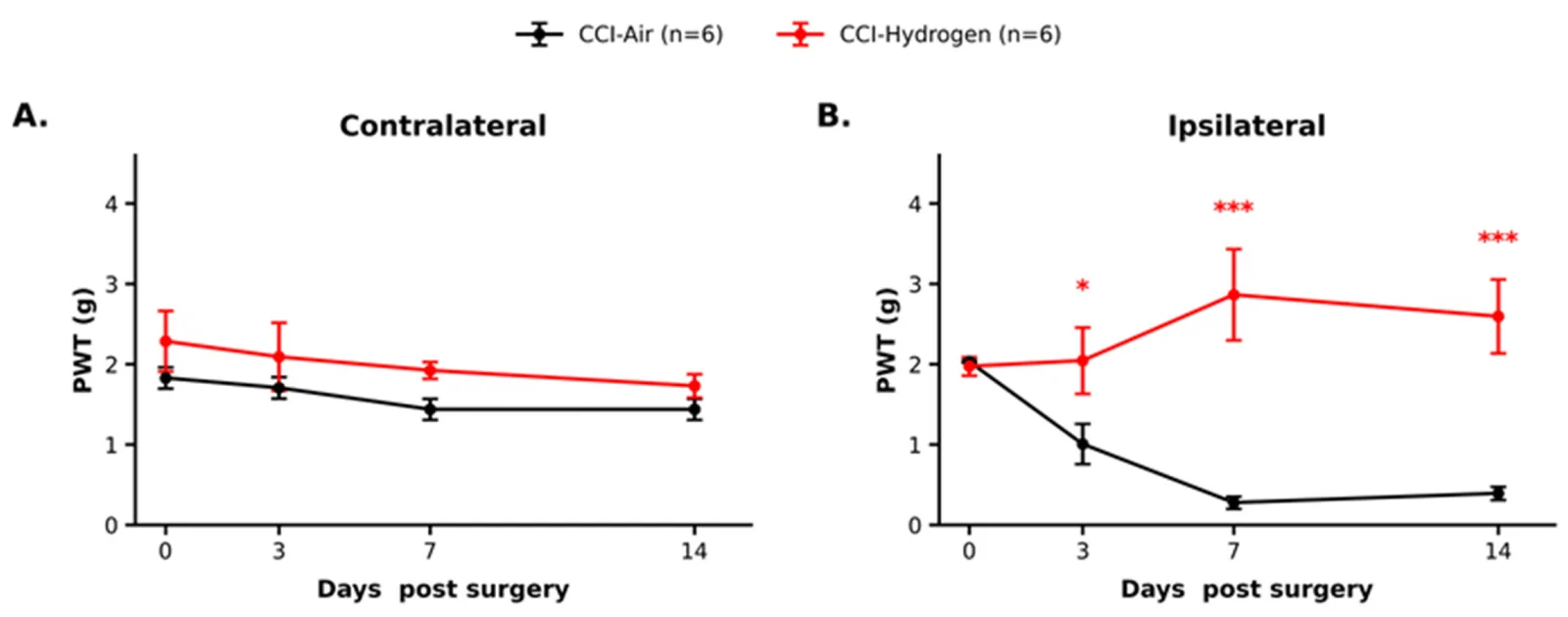
Exploratory murine chronic constriction injury (CCI) experiment. Paw withdrawal threshold (PWT) was measured in contralateral (**A**) and ipsilateral (**B**) hind paws at baseline (day 0) and on days 3, 7, and 14 after CCI. Data are presented as mean ± standard error of the mean (SEM); CCI-Air, *n* = 6; CCI-Hydrogen, *n* = 6. Data were analyzed by GEE accounting for repeated measurements within each animal, with Holm-adjusted hydrogen-minus-air contrasts at each postoperative day. * Holm-adjusted *p* < 0.05; *** Holm-adjusted *p* < 0.001 for the between-group difference on that day. The contralateral limb was not ligated; the ipsilateral limb was the CCI-treated side.

For the ipsilateral (CCI-treated) paw, the group-by-day interaction was significant (Wald χ^2^(3) = 36.11, *p* < 0.001), indicating that the trajectory of mechanical thresholds differed between groups (Figure 3B). Baseline thresholds were comparable (day 0, hydrogen-minus-air difference −0.06 g; 95% CI −0.27 to 0.15; *p* = 0.576). After CCI, air-control mice developed progressive ipsilateral allodynia (from 2.03 ± 0.04 g at day 0 to 0.39 ± 0.20 g at day 14), whereas hydrogen-treated animals maintained higher thresholds: +1.04 g (95% CI 0.18 to 1.90; Holm-adjusted *p* = 0.018) at day 3, +2.59 g (95% CI 1.56 to 3.61; Holm-adjusted *p* < 0.001) at day 7, and +2.20 g (95% CI 1.37 to 3.04; Holm-adjusted *p* < 0.001) at day 14. In the contralateral uninjured paw, the group-by-day interaction was not significant (Wald χ^2^(3) = 0.72, *p* = 0.869), and thresholds changed little over time in either group (Figure 3A). Because hydrogen exposure began on the day of CCI surgery, this experiment evaluated prevention/attenuation of allodynia development and did not test whether hydrogen reverses established allodynia.

## 4. Discussion

The principal clinical finding of this pilot study is a difference in longitudinal postoperative pain trajectories between the hydrogen and control cohorts. The significant group-by-time interaction (Wald χ^2^(6) = 56.68, *p* < 0.001) supports an overall difference in the pattern of pain-score change over time. After multiplicity adjustment, the clearest time-specific differences were observed at PACU arrival, 1 h, and 1 month. Lower intraoperative fentanyl consumption and less frequent rescue analgesic use were directionally concordant with the pain-score findings.

Statistically, the significant group-by-time interaction means that the two cohorts did not follow the same longitudinal pain-score pattern. The time point-specific contrasts therefore serve to localize and quantify the overall trajectory difference. The estimated H_2_-minus-control differences were −3.21 points at PACU and −2.42 points at 1 h, with both remaining significant after Holm adjustment. At 6 and 12 h, the estimated differences remained in the same direction (−1.81 and −1.72 points, respectively) but did not reach statistical significance (*p* values were 0.071 and 0.081, respectively). A plausible explanation for an analgesic effect of molecular hydrogen is modulation of redox-sensitive inflammatory and neuroimmune pathways that contribute to peripheral nociceptor sensitization and central sensitization. Foundational experimental work showed that H_2_ can reduce highly reactive oxygen species and limit oxidative injury [14], while subsequent studies have linked hydrogen exposure to broader modulation of inflammatory signaling [15,16]. More directly relevant to postoperative pain, Li et al. demonstrated in a plantar-incision model that H_2_ increased Trx1 expression, inhibited ASK1/p38/JNK phosphorylation and MMP-9 activity, reduced IL-1β maturation and microglial activation, and attenuated mechanical allodynia [21]. Incisional pain also engages TNF-α/p38 MAPK/NF-κB and sodium-channel pathways [28]. In neuropathic pain models, hydrogen-containing interventions have likewise been associated with reductions in oxidative stress, glial activation, and inflammatory cytokine signaling, together with improvement in mechanical allodynia [18,20]. These findings make an inflammation- and redox-related antinociceptive mechanism biologically plausible, but the present clinical study did not directly measure spinal signaling, nociceptor sensitization, or a validated mediator pathway and therefore cannot identify a single mechanism of action.

The temporal pattern of the clinical findings requires a more nuanced interpretation than a simple present-versus-absent analgesic effect. The largest early between-group differences occurred at PACU arrival and 1 h immediately after an intraoperative H_2_ exposure that ended at surgical completion. This temporal proximity is compatible with an acute biological effect of H_2_ but does not establish causality, particularly because treatment was not randomized or blinded. Thereafter, both groups received the same postoperative multimodal analgesic strategy, while surgical decompression itself would be expected to reduce a component of pre-existing radicular pain. In addition, H_2_-group pain scores were already approximately 2–3 points during much of the first postoperative day, leaving limited room for further reduction, whereas pain in the control group progressively declined. Together with the small sample and multiplicity correction, these factors can plausibly account for the less certain differences at 6 and 12 h and the substantial convergence by 24 h. Accordingly, the lack of adjusted statistical significance at 6 and 12 h should not be interpreted as evidence of a biologically abrupt loss of effect.

The renewed between-group difference at 1 month should be interpreted separately from the immediate postoperative findings. Human inhalation data indicate that blood H_2_ concentrations fall rapidly after inhalation is discontinued [29]. In the present cohort, the late contrast was driven largely by an increase in control-group pain between 24 h and 1 month, whereas H_2_-group scores remained around 2–3 points. Several non-exclusive explanations are possible, including re-emergence of residual radicular symptoms after acute perioperative analgesia had resolved, differences in recovery trajectories, longer-lasting consequences of early inflammatory modulation, temporal confounding inherent in the sequential cohort design, or simple sampling variability. The 1-month finding should therefore be considered hypothesis-generating rather than evidence that a single perioperative H_2_ exposure has a persistent direct analgesic action. The exploratory PBMC data are within-group observations from H_2_-treated participants only and are presented in the Supplementary Materials. Because no control samples were obtained and surgery itself suppresses ex vivo cytokine responses [30], they cannot be attributed to hydrogen and serve only to motivate a controlled study.

The murine CCI experiment provides an additional and independent line of biological plausibility, particularly for the neuropathic component of the clinical pain phenotype. In a repeated-measures analysis, the trajectory of ipsilateral withdrawal thresholds differed significantly between groups, with hydrogen-treated mice maintaining higher thresholds on days 3, 7, and 14 despite comparable baseline values, whereas the contralateral uninjured paw showed no such divergence. This pattern is directionally consistent with previous neuropathic-pain experiments in which molecular hydrogen or hydrogen-rich preparations attenuated allodynia together with reductions in oxidative stress, glial activation, and inflammatory signaling [18,20]. Nevertheless, the murine and human components do not represent direct replication of the same pain phenotype. CCI produces a standardized peripheral neuropathic injury, whereas pain after endoscopic discectomy likely combines acute incisional nociception, postoperative inflammation, and residual or resolving radicular/neuropathic pain. Exposure paradigms were also markedly different: mice began H_2_ on the day of nerve injury and received treatment daily for 14 days, whereas patients received only intraoperative exposure. Moreover, the animal control was not oxygen-matched: because the delivered mixture contained 33.3% O_2_ while controls breathed ambient air at approximately 21% O_2_, hydrogen-treated mice were simultaneously exposed to a higher oxygen fraction, and directly documented chamber H_2_/O_2_ concentrations were unavailable.

Lower intraoperative fentanyl consumption and less rescue analgesic use accompanied the lower pain scores, and this concordance strengthens the internal consistency of the observation. Because treatment assignment was not blinded, however, these analgesic outcomes may be influenced by expectation effects, and a confirmatory trial should use blinded allocation with a prespecified analgesic protocol.

No adverse events were documented among the H_2_-treated participants. This observation is nonetheless consistent with the broader safety profile of inhaled molecular hydrogen. Hydrogen is not metabolized to a toxic intermediate and has no known dose-limiting organ toxicity at the concentrations used therapeutically. Prolonged inhalation in healthy adults and inhalation in acute stroke patients monitored with serial physiological measurements have not produced clinically significant hemodynamic, respiratory, or laboratory abnormalities [29,31]. Perioperative administration has likewise been tolerated without major physiological instability in a controlled large-animal model of circulatory arrest [32]. In the present study, the generator produced a 66.7% H_2_/33.3% O_2_ source gas delivered at 2 L/min through an open nasal cannula, while the approximately 4% inspired H_2_ concentration was a nominal estimate based on assumed dilution with ambient air rather than direct measurement. Thus, actual patient-level FiH_2_ and FiO_2_ were uncertain, which limits both dose reproducibility and safety characterization.

The flammability hazard is best considered by distinguishing room-level from local concentrations. At the room level, the margin is substantial. Hydrogen was delivered at approximately 1.33 L/min (0.08 m^3^/h), and operating rooms are mechanically ventilated at a minimum of 20 air changes per hour under ANSI/ASHRAE/ASHE Standard 170, so that the delivered gas is continuously removed rather than permitted to accumulate. An entire hour of hydrogen output would have to be confined and completely mixed within approximately 2 m^3^ of air to reach the 4 vol% lower flammability limit of hydrogen in air, a volume considerably smaller than that of a standard operating room. The low density of hydrogen further favors upward dispersion toward the ceiling exhaust rather than accumulation over the surgical field, and because the source gas is generated on demand by water electrolysis, no pressurized hydrogen is stored within the room.

The residual hazard is local, at the cannula, where the undiluted mixture remains within the flammable range and proximity to an ignition source, rather than systemic toxicity, is the principal consideration. Reported hydrogen-inhaler incidents have been attributed largely to gas leakage within or around the device [33]. This is the same fuel–oxidizer–ignition problem that operating rooms already manage for oxygen-enriched atmospheres, and the established preventive framework therefore applies directly [34]: maintaining separation between the gas source and electrosurgical or laser devices, avoiding drape pockets or other enclosed spaces near the head in which gas could accumulate, leak-testing tubing and connections before use, and interrupting delivery if electrosurgery is required close to the face. The procedure studied here is favorable in this respect, since the patient is prone and the lumbar surgical field is remote from the nasal cannula; the same arrangement should not be assumed transferable to head, neck, or airway surgery, in which the ignition source and the gas source would be adjacent [34].

In the present study, hydrogen was administered under the standard institutional fire-safety practices governing all procedures in our operating rooms, and no adverse or combustion events occurred in the analyzed cohort. Ambient operating-room hydrogen concentrations were not monitored during the study, so measured environmental exposure cannot be reported. These observations therefore support the tolerability of this delivery approach but do not substitute for prospective safety evaluation. Future trials should directly measure inspired and ambient H_2_ concentrations and prespecify hydrogen-specific procedures, including leak detection and continuous ambient monitoring with an alarm threshold set at a defined fraction of the lower flammability limit.

Several limitations substantially constrain causal interpretation. First, treatment assignment was determined by two consecutive enrollment periods rather than randomization, and neither participants nor clinicians were blinded. Although the enrollment periods were temporally adjacent, period-based allocation permits selection, performance, expectation, and temporal confounding. Second, complete source-verifiable outcome records were unavailable for 12 of the 25 participants enrolled during the H_2_ period. Excluding unverifiable reconstructed data was methodologically conservative, but because all such exclusions occurred in one treatment cohort, selection bias cannot be excluded, and the direction or magnitude of that bias cannot be determined from the available data. Third, only 25 independent participants contributed to the longitudinal analysis. GEE with robust covariance accounts for within-participant correlation, but robust Wald inference remains asymptotic and should therefore be interpreted cautiously in a sample with a small number of independent subjects. Fourth, the NRS score did not distinguish acute incisional nociception from residual radicular or neuropathic pain and remained vulnerable to expectation effects in an unblinded study. Fifth, the registered 1-week assessment was unavailable in the source-verifiable dataset, limiting interpretation of the apparent re-divergence between 24 h and 1 month. Sixth, the actual inspired H_2_ concentration was not measured, and ambient operating-room hydrogen was not monitored. Seventh, the PBMC analysis lacked contemporaneous control samples and cannot identify a hydrogen-specific anti-inflammatory effect. Eighth, the murine experiment differed from the clinical study in pain phenotype and exposure schedule, used a control exposure that was not oxygen-matched, so that hydrogen-treated mice also received a higher oxygen fraction, and used only male animals. Because hydrogen was administered from the day of nerve injury, the murine experiment tested prevention of the development of mechanical allodynia rather than reversal of established allodynia, and whether hydrogen is effective once neuropathic pain is established remains untested. Collectively, these limitations require the clinical and animal findings to be regarded as internally and biologically concordant but preliminary, with the immune observations treated as descriptive only.

The appropriate next step is a prospectively randomized, allocation-concealed, double-blind, sham-controlled trial. Such a study should use an oxygen-matched and visually indistinguishable control delivery system, prospectively characterize actual FiH_2_/FiO_2_ and ambient H_2_ exposure, standardize perioperative opioid and non-opioid analgesia, prespecify the longitudinal estimand and multiplicity strategy, and include validated instruments that distinguish neuropathic/radicular pain from acute postoperative nociception and functional recovery [35]. Serial blood sampling should be obtained at identical time points in both groups with prespecified inflammatory and redox biomarkers so that associations among H_2_ exposure, biomarker modulation, opioid requirement, and pain can be formally tested rather than inferred from parallel observations. Particular attention should be given to reproducing the early PACU/1-h signal and determining whether the unexpected 1-month difference persists in a randomized population.

## 5. Conclusions

In this small, open-label, non-randomized pilot study, perioperative hydrogen inhalation was associated with a different longitudinal postoperative pain trajectory after endoscopic discectomy; after multiplicity adjustment, the clearest between-group differences were observed at PACU arrival, 1 h, and 1 month, accompanied by lower intraoperative fentanyl consumption and less rescue analgesic use. The murine CCI experiment provided directionally consistent biological plausibility. However, period-based treatment allocation, differential loss of source-verifiable H_2_ records, small sample size, unblinded treatment, unmeasured inspired H_2_ concentration, and differences between the animal and clinical phenotypes and exposure regimens preclude causal inference regarding efficacy or mechanism. The present findings should therefore be considered hypothesis-generating and require confirmation in a randomized, blinded, sham-controlled trial with standardized hydrogen delivery, protocolized analgesia, prespecified longitudinal analysis, and balanced mechanistic biomarker sampling.

## Figures and Tables

**Table 1:** Baseline characteristics and perioperative outcomes among the 25 patients included in the source-verifiable complete-case analysis.

	Hydrogen Inhalation (*n* = 13)	Control Group (*n* = 12)	*p* Value
Male sex, *n* (%)	5 (38.46)	4 (33.33)	1.000
Age in years (SD)	61.31 (13.60)	68.5 (17.35)	0.259
Comorbidities			
Hypertension, *n* (%)	7 (53.85)	9 (75.00)	0.411
Diabetes mellitus, *n* (%)	3 (23.07)	2 (16.67)	1.000
Cardiac disease, *n* (%)	2 (15.38)	5 (41.67)	0.202
Liver and renal disease, *n* (%)	1 (7.69)	1 (8.33)	1.000
Smoking, *n* (%)	1 (7.69)	3 (25.00)	0.322
Right side, *n* (%)	8 (61.53)	8 (66.67)	1.000
Spine level			
L2/3, *n* (%)	0 (0.00)	1 (8.33)	
L3/4, *n* (%)	2 (15.38)	4 (33.33)	
L4/5, *n* (%)	11 (84.61)	7 (58.33)	0.251
Inhalation time in minutes (SD)	249.62 (38.96)	241.17 (37.86)	0.588
Intraoperative fentanyl in μg (SD)	265.38 (99.24)	347.92 (93.82)	0.044 *
Rescue analgesic use, *n* (%)	5 (38.46)	10 (83.33)	0.041 *

SD: standard deviation. Fisher’s exact test was used for sparse 2 × 2 categorical comparisons; an exact contingency-table procedure was used for the multilevel spinal-level comparison. * Statistically significant (*p* < 0.05).

**Table 2:** GEE-derived between-group contrasts in longitudinal NRS pain scores.

Assessment Time	Hydrogen Mean ± SD	Control Mean ± SD	H_2_-Control Mean Difference	95% Wald CI	Holm-Adjusted *p*
Preoperative	8.15 ± 1.07	8.17 ± 1.03	−0.01	−0.80 to 0.78	0.975 ^†^
PACU	2.04 ± 1.76	5.25 ± 2.86	−3.21	−5.02 to −1.41	0.002
1 h	2.50 ± 1.83	4.92 ± 1.98	−2.42	−3.85 to −0.98	0.004
6 h	2.27 ± 2.07	4.08 ± 2.11	−1.81	−3.39 to −0.24	0.071
12 h	2.12 ± 2.16	3.83 ± 2.21	−1.72	−3.36 to −0.07	0.081
24 h	2.92 ± 1.89	3.67 ± 2.23	−0.74	−2.30 to 0.82	0.350
1 month	2.38 ± 1.94	5.83 ± 1.64	−3.45	−4.80 to −2.10	<0.001

Mean differences are GEE-derived hydrogen-minus-control contrasts; negative values favor hydrogen. The six postoperative *p* values were adjusted using the Holm step-down procedure. The reported 95% Wald CIs were not multiplicity-adjusted. ^†^ The preoperative comparison was not included in the six-comparison postoperative Holm family.

## Data Availability

The de-identified, source-verifiable data supporting the 25-patient clinical analysis are available on request from the corresponding author because of participant privacy and institutional data-sharing restrictions. The 12 excluded participants are not part of the analytic dataset. Individual-level animal data for the chronic constriction injury experiment were subsequently retrieved from the laboratory archive and used for the repeated-measures analysis reported in this revision. These data are available upon request from the corresponding author.

## Supplementary Materials

The following supporting information can be downloaded at https://www.mdpi.com/article/10.3390/medicina62091785/s1, Supplementary Methods S1. Exploratory PBMC analysis; Supplementary Results S1. Exploratory inflammatory responsiveness; Table S1. Exploratory PBMC cytokine concentrations in hydrogen-treated participants (*n* = 13). Figure S1. Exploratory PBMC cytokine secretion before and after surgery in hydrogen-treated participants. LPS-stimulated supernatant concentrations of (A) TNF-α, (B) IFN-γ, (C) IL-1β, and (D) IL-10 in paired preoperative and postoperative samples (*n* = 13). Grey lines show individual participants; red symbols show mean ± SEM. P values are from two-tailed paired Student *t* tests and are unadjusted and are shown to describe the within-group change rather than to test a hypothesis. Control-group samples were not obtained, so these data are descriptive and no causal inference is intended.

## Abbreviations

The following abbreviations are used in this manuscript:

H_2_	Molecular hydrogen
ROS	Reactive oxygen species
CCI	Chronic constriction injury
PBMC	Peripheral blood mononuclear cell
NRS	Numeric rating scale
GEE	Generalized estimating equation
ASA	American Society of Anesthesiologists
PACU	Post-anesthesia care unit
IACUC	Institutional Animal Care and Use Committee
NRS	Numerical rating scale
CI	Confidence interval
SD	Standard deviation
SEM	Standard error of the mean
PWT	Paw withdrawal threshold
NF-κB	Nuclear factor kappa-light-chain-enhancer of activated B cells

## References

[1] Baron R., Binder A., Attal N., Casale R., Dickenson A.H., Treede R. (2016). Neuropathic low back pain in clinical practice. Eur. J. Pain.

[2] Berry J.A., Elia C., Saini H.S., Miulli D.E. (2019). A Review of Lumbar Radiculopathy, Diagnosis, and Treatment. Cureus.

[3] Ahn Y. (2025). Strategies to Reduce Rebound Pain and Facilitate Early Recovery After Transforaminal Endoscopic Lumbar Discectomy. J. Clin. Med..

[4] Hara T., Ohara Y. (2023). Perioperative Management for Full-Endoscopic Lumbar Discectomy: Consideration From the Perspective of Preventing Complication. Neurospine.

[5] Lin G.X., Sun L.W., Jhang S.W., Chen C.M., Rui G., Hu B.S. (2022). Postoperative Pain Management after Full Endoscopic Lumbar Discectomy: An Observational Study. Medicina.

[6] Savic Vujovic K., Zivkovic A., Dozic I., Cirkovic A., Medic B., Srebro D., Vuckovic S., Milovanovic J., Jotic A. (2023). Oxidative Stress and Inflammation Biomarkers in Postoperative Pain Modulation in Surgically Treated Patients with Laryngeal Cancer—Pilot Study. Cells.

[7] Carrasco C., Nazıroğlu M., Rodríguez A.B., Pariente J.A. (2018). Neuropathic Pain: Delving into the Oxidative Origin and the Possible Implication of Transient Receptor Potential Channels. Front. Physiol..

[8] Yowtak J., Lee K.Y., Kim H.Y., Chung K., Chung J.M. (2011). Reactive oxygen species contribute to neuropathic pain by reducing spinal GABA release. Pain.

[9] Kim H.K., Park S.K., Zhou J.L., Taglialatela G., Chung K., Coggeshall R.E., Chung J.M. (2004). Reactive oxygen species (ROS) play an important role in a rat model of neuropathic pain. Pain.

[10] Gao X., Kim H.K., Mo Chung J., Chung K. (2007). Reactive oxygen species (ROS) are involved in enhancement of NMDA-receptor phosphorylation in animal models of pain. Pain.

[11] Chung J.M. (2004). The role of reactive oxygen species (ROS) in persistent pain. Mol. Interv..

[12] Hartung J.E., Eskew O., Wong T., Tchivileva I.E., Oladosu F.A., O’bUckley S.C., Nackley A.G. (2015). Nuclear factor-kappa B regulates pain and COMT expression in a rodent model of inflammation. Brain Behav. Immun..

[13] Fu E.S., Zhang Y.P., Sagen J., Candiotti K.A., Morton P.D., Liebl D.J., Bethea J.R., Brambilla R. (2010). Transgenic Inhibition of Glial NF-kappa B Reduces Pain Behavior and Inflammation after Peripheral Nerve Injury. Pain.

[14] Ohsawa I., Ishikawa M., Takahashi K., Watanabe M., Nishimaki K., Yamagata K., Katsura K.-I., Katayama Y., Asoh S., Ohta S. (2007). Hydrogen acts as a therapeutic antioxidant by selectively reducing cytotoxic oxygen radicals. Nat. Med..

[15] Tian Y., Zhang Y., Wang Y., Chen Y., Fan W., Zhou J., Qiao J., Wei Y. (2021). Hydrogen, a Novel Therapeutic Molecule, Regulates Oxidative Stress, Inflammation, and Apoptosis. Front. Physiol..

[16] Ren J.D., Ma J., Hou J., Xiao W.-J., Jin W.-H., Wu J., Fan K.-H. (2014). Hydrogen-rich saline inhibits NLRP3 inflammasome activation and attenuates experimental acute pancreatitis in mice. Mediat. Inflamm..

[17] Chen Q., Chen P., Zhou S., Yan X., Zhang J., Sun X., Yuan H., Yu W. (2013). Hydrogen-rich saline attenuated neuropathic pain by reducing oxidative stress. Can. J. Neurol. Sci..

[18] Kawaguchi M., Satoh Y., Otsubo Y., Kazama T. (2014). Molecular hydrogen attenuates neuropathic pain in mice. PLoS ONE.

[19] Martínez-Serrat M., Martínez-Martel I., Coral-Pérez S., Bai X., Batallé G., Pol O. (2022). Hydrogen-Rich Water as a Novel Therapeutic Strategy for the Affective Disorders Linked with Chronic Neuropathic Pain in Mice. Antioxidants.

[20] Ge Y., Wu F., Sun X., Xiang Z., Yang L., Huang S., Lu Z., Sun Y., Yu W.-F. (2014). Intrathecal Infusion of Hydrogen-Rich Normal Saline Attenuates Neuropathic Pain via Inhibition of Activation of Spinal Astrocytes and Microglia in Rats. PLoS ONE.

[21] Li J., Ruan S., Jia J., Li Q., Jia R., Wan L., Yang X., Teng P., Peng Q., Shi Y.-D. (2023). Hydrogen attenuates postoperative pain through Trx1/ASK1/MMP9 signaling pathway. J. Neuroinflamm..

[22] Lancaster G.A., Thabane L. (2019). Guidelines for reporting non-randomised pilot and feasibility studies. Pilot Feasibility Stud..

[23] Percie du Sert N., Hurst V., Ahluwalia A., Alam S., Avey M.T., Baker M., Browne W.J., Clark A., Cuthill I.C., Dirnagl U. (2020). The ARRIVE guidelines 2.0: Updated guidelines for reporting animal research. PLoS Biol..

[24] Bennett G.J., Xie Y.K. (1988). A peripheral mononeuropathy in rat that produces disorders of pain sensation like those seen in man. Pain.

[25] Kung C.C., Dai S.P., Yen C.H., Lee Y.-J., Chang S.-L., Fang Y.-T., Lin H.-L., Chen C.-L. (2025). Animal neuropathic pain aroused by conglutinating oxidative regenerative cellulose on dorsal root ganglion. J. Neuropathol. Exp. Neurol..

[26] Liang K.Y., Zeger S.L. (1986). Longitudinal data analysis using generalized linear models. Biometrika.

[27] Holm S. (1979). A simple sequentially rejective multiple test procedure. Scand. J. Stat..

[28] de Lima F.O., Lauria P.S.S., do Espírito-Santo R.F., Evangelista A.F., Nogueira T.M.O., Araldi D., Soares M.B.P., Villarreal C.F. (2022). Unveiling Targets for Treating Postoperative Pain: The Role of the TNF-α/p38 MAPK/NF-κB/Nav1.8 and Nav1.9 Pathways in the Mouse Model of Incisional Pain. Int. J. Mol. Sci..

[29] Ono H., Nishijima Y., Adachi N., Sakamoto M., Kudo Y., Kaneko K., Nakao A., Imaoka T. (2012). A basic study on molecular hydrogen (H2) inhalation in acute cerebral ischemia patients for safety check with physiological parameters and measurement of blood H2 level. Med. Gas Res..

[30] Kawasaki T., Ogata M., Kawasaki C., Tomihisa T., Okamoto K., Shigematsu A. (2001). Surgical stress induces endotoxin hyporesponsiveness and an early decrease of monocyte mCD14 and HLA-DR expression during surgery. Anesth. Analg..

[31] Cole A.R., Sperotto F., DiNardo J.A., Carlisle S., Rivkin M.J., Sleeper L.A.S., Kheir J.N. (2021). Safety of Prolonged Inhalation of Hydrogen Gas in Air in Healthy Adults. Crit. Care Explor..

[32] Cole A.R., Perry D.A., Raza A., Nedder A.P., Pollack E., Regan W.L., Bosch S.J.v.D., Polizzotti B.D., Yang E., Davila D. (2019). Perioperatively Inhaled Hydrogen Gas Diminishes Neurologic Injury Following Experimental Circulatory Arrest in Swine. JACC Basic Transl. Sci..

[33] Ichikawa Y., Hirano S., Sato B., Yamamoto H., Takefuji Y., Satoh F. (2023). Guidelines for the selection of hydrogen gas inhalers based on hydrogen explosion accidents. Med. Gas Res..

[34] Apfelbaum J.L., Caplan R.A., Barker S.J., Connis R.T., Cowles C., Ehrenwerth J., Nickinovich D.G., Pritchard D., Roberson D.W., de Richemond A.L. (2013). Practice Advisory for the Prevention and Management of Operating Room Fires: An Updated Report by the American Society of Anesthesiologists Task Force on Operating Room Fires. Anesthesiology.

[35] Cruccu G., Truini A. (2009). Tools for Assessing Neuropathic Pain. PLoS Med..

