# Supplementary material for: Perioperative Hydrogen Inhalation and Postoperative Pain After Endoscopic Discectomy: A Pilot Non-Randomized Clinical Study with Exploratory Murine Data
Source: Medicina (Kaunas). 2026 Sep 16;62(9):1785. doi: 10.3390/medicina62091785 (PMC13609074; doi:10.3390/medicina62091785)
Supplement: Supplementary file 1 [file medicina-62-01785-s001.zip › medicina-4425004-supplementary.pdf]

Supplementary Materials

Perioperative Hydrogen Inhalation and Postoperative Pain after Endoscopic Discectomy

Supplementary Methods S1. Exploratory PBMC analysis

Sample collection. Paired peripheral blood samples were obtained from hydrogen-treated participants immediately before and immediately after surgery. No blood samples were collected from control-group participants; this component was therefore a within-group, pre-post analysis and was not designed for between-group comparison. Whole blood (12–20 mL) was drawn into lithium-heparin tubes. Personnel performing the isolation, culture, and assays were blinded to sample identity. IL-6 was registered to be tested but was not performed. We performed analysis on TNF-α, IFN-γ, IL-10 and IL-1β.

PBMC isolation and culture. PBMCs were isolated by Percoll PLUS (Cytiva, Marlborough, MA, USA) density-gradient centrifugation at 2500 rpm for 45 min at room temperature. Isolated mononuclear cells were washed with 0.15 M NaCl and resuspended at 2×10^6 cells/mL in RPMI 1640 medium supplemented with 10% fetal bovine serum and 1% penicillin–streptomycin. Cell viability was assessed by Trypan blue exclusion, and cell counts were determined with a Neubauer chamber. Cells were plated in 24-well plates (1 mL per well) and stimulated with lipopolysaccharide (LPS, 1 μg/mL; Sigma-Aldrich) and RPMI 1640 medium alone as a vehicle control. Each condition was run in three biological replicates and incubated for 48 h at 37 °C in a humidified atmosphere containing 5% CO_2. Culture supernatants were collected, centrifuged at 1500 rpm for 5 min at 4 °C, and stored at −80 °C until analysis.

Cytokine quantification. Supernatant concentrations of TNF-α (Sino Biological, #SEKA10602, Beijing, China), IL-1β (R&D Systems, #DY201-05, Minneapolis, MN, USA), IFN-γ (Invitrogen, #88-7316, Waltham, MA, USA), and IL-10 (R&D Systems, #DY217B-05, Minneapolis, MN, USA) were measured by enzyme-linked immunosorbent assay according to the manufacturers' instructions. The results reported here are those obtained under LPS stimulation.

Statistical analysis. Preoperative and postoperative concentrations were compared within participants using two-tailed paired Student t tests, and data are presented as mean ± standard error of the mean (SEM). Because no contemporaneous control samples were available, this component is descriptive and no inferential claim is made about a hydrogen-specific effect. P values are therefore presented unadjusted and should be read as descriptive indices of within-group change rather than as hypothesis tests; they are not corrected for multiplicity across the four cytokines, and the four cytokines are not treated as a single family of tests, since each was measured as a distinct biological readout rather than as an alternative measure of the same outcome.

Supplementary Results S1. Exploratory inflammatory responsiveness

Paired pre- and postoperative samples were available from 13 hydrogen-treated participants. In LPS-stimulated PBMC cultures, postoperative secretion of the pro-inflammatory cytokines TNF-α, IFN-γ, and IL-1β was lower than the corresponding preoperative response, with unadjusted paired P values of 0.043, 0.016, and 0.026, respectively (Table S1, Figure S1). Secretion of the anti-inflammatory cytokine IL-10 also declined, by a comparable relative magnitude, but this change did not reach conventional statistical significance ($P = 0.081$).

These findings should be interpreted with several constraints in mind. First, no control-group samples were obtained, and surgery and anaesthesia alone are known to suppress ex-vivo cytokine

responsiveness; the observed within-group changes are therefore compatible with hydrogen exposure, with the postoperative immune response itself, or with both, and cannot be attributed specifically to hydrogen. Second, the non-significant IL-10 result should not be read as evidence that anti-inflammatory signaling was preserved or that immunosuppression was absent: IL-10 declined in the same direction and to a similar relative degree as the pro-inflammatory cytokines, and the analysis was not powered to demonstrate a difference between cytokine classes. Third, the P values are unadjusted and are reported only to describe the magnitude and consistency of the within-group change; with 13 participants and four separate readouts they should not be used to support any inferential claim. These data are presented as descriptive, hypothesis-generating observations that motivate a randomized study with balanced serial sampling in both groups.

Table S1. Exploratory PBMC cytokine concentrations in hydrogen-treated participants (n = 13)

Cytokine	Preoperative (pg/mL)	Postoperative (pg/mL)	Relative change	P value
TNF-α	1776 ± 232	1326 ± 158	−25.3%	0.043
IFN-γ	467 ± 140	150 ± 46	−67.9%	0.016
IL-1β	1220 ± 174	907 ± 126	−25.6%	0.026
IL-10	2281 ± 402	1586 ± 237	−30.5%	0.081

Values are mean ± SEM of LPS-stimulated culture supernatant concentrations. P values are from two-tailed paired Student t tests and are unadjusted; they are descriptive and are not intended as hypothesis tests. PBMC: peripheral blood mononuclear cell; LPS: lipopolysaccharide; SEM: standard error of the mean. No control-group samples were available; this table is descriptive and does not demonstrate a hydrogen-specific effect.

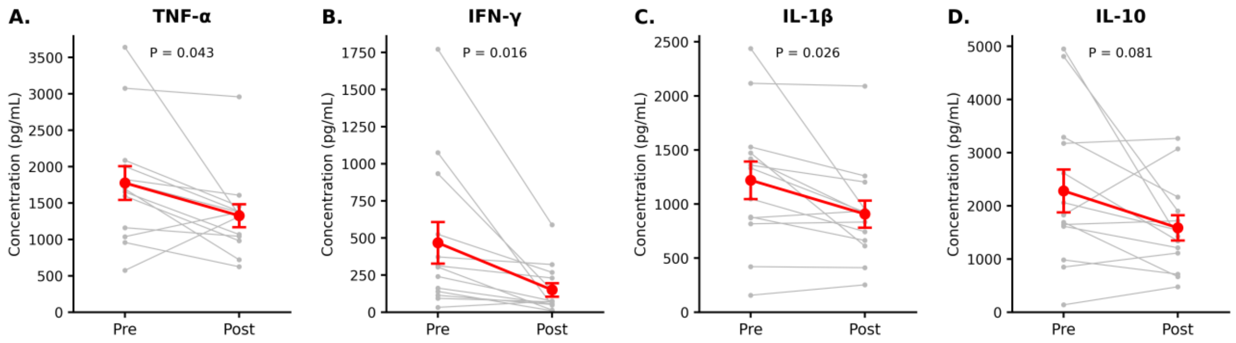

Figure S1. Exploratory PBMC cytokine secretion before and after surgery in hydrogen-treated participants. LPS-stimulated supernatant concentrations of (A) TNF-α, (B) IFN-γ, (C) IL-1β, and (D) IL-10 in paired preoperative and postoperative samples (n = 13). Grey lines show individual participants; red symbols show mean ± SEM. P values are from two-tailed paired Student t tests and are unadjusted and are shown to describe the within-group change rather than to test a hypothesis. Control-group samples were not obtained, so these data are descriptive and no causal inference is intended.
